# Asian expert consensus on high-quality hypertension management

**DOI:** 10.1038/s41440-026-02644-2

**Published:** 2026-05-12

**Authors:** Jing Liu, Kazuomi Kario, Xiaoqiang Ding, Zixiao Li, Tianli Gao, Yan Li, Yuqing Zhang, Wei Chen, Yook-Chin Chia, Yuhua Fan, Satoshi Hoshide, Huijuan Mao, Jinho Shin, Apichard Sukonthasarn, Boon Wee Teo, Yuda Turana, Li Yao, Hong Zhang, Ji-Guang Wang

**Affiliations:** 1 Department of Hypertension, Peking University People’s Hospital; Vascular Health Research Center, Peking University Health Science Center, Beijing, China; 2 Division of Cardiovascular Medicine, Department of Medicine, Jichi Medical University School of Medicine, Tochigi, Japan; 3 Nephrology Department, Zhongshan Hospital Affiliated to Fudan University, Shanghai, China; 4 Department of Neurology, Beijing Tiantan Hospital Affiliated to Capital Medical University, Beijing, China; 5 Department of Cerebrovascular Disease, Beijing Anzhen Hospital Affiliated to Capital Medical University, Beijing, China; 6 Department of Cardiovascular Medicine, Shanghai Institute of Hypertension, Shanghai Key Laboratory of Hypertension, State Key Laboratory of Medical Genomics, National Research Centre for Translational Medicine at Shanghai, Ruijin Hospital, Shanghai Jiaotong University School of Medicine, Shanghai, China; 7 Divisions of Hypertension and Heart Failure, Fu Wai Hospital, Chinese Academy of Medical Sciences and Peking Union Medical College, Beijing, China; 8 Department of Nephrology, The First Affiliated Hospital of Sun Yat-sen University, Guangzhou, China; 9 Department of Clinical Medicine and Surgery Sir Jeffrey Cheah Sunway Medical School, Faculty of Medical and Life Sciences, Sunway University, Bandar Sunway, Selangor, Malaysia; Department of Primary Care Medicine, Faculty of Medicine, Universiti Malaya, Kuala Lumpur, Malaysia; Ageing, Health and Wellbeing Research Centre (CARE), Faculty of Medical and Life Sciences, Sunway University, Bandar Sunway, Selangor, Malaysia; 10 Department of Neurology, The First Affiliated Hospital of Sun Yat-sen University, Guangzhou, China; 11 Department of Nephrology, Jiangsu Province Hospital (The First Affiliated Hospital with Nanjing Medical University), Nanjing, China; 12 Faculty of Cardiology Service, Hanyang University Medical Center, Seoul, South Korea; 13 Department of Medicine, Faculty of Medicine, Chiang Mai University, Chiang Mai, Thailand; 14 Division of Nephrology Department of Medicine, Yong Loo Lin School of Medicine, National University of Singapore, Singapore, Singapore; 15 Department of Neurology. School of Medicine and Health Sciences, Atma Jaya Catholic University of Indonesia, Jakarta, Indonesia; 16 Department of Nephrology, The First Affiliated Hospital of China Medical University, Shenyang, China; 17 Renal Division, Peking University First Hospital, Beijing, China; 18 Department of Hypertension, Centre for Epidemiological Studies and Clinical Trials, the Shanghai Institute of Hypertension, Shanghai Key Laboratory of Hypertension, State Key Laboratory of Medical Genomics, Ruijin Hospital, Shanghai Jiaotong University School of Medicine, Shanghai, China

**Keywords:** 24-h blood pressure control, High-quality hypertension management, Implemention hypertension, Long term blood pressure management, Time-in-target range

## Abstract

High-quality hypertension management is a new concept proposed to improve blood pressure (BP) control in Asia. Out-of-office BP measurements, including ambulatory and home BP monitoring, and wearable BP measurement, are recommended for BP assessment. Long-acting antihypertensive agents at full dose or in combination are priority strategies for achieving 24-h (24-h) BP control, reducing blood pressure variability (BPV) and improving time-in-target range (TTR). High-quality hypertension management across multiple disciplines will be a pragmatic strategy for the reduction in the risk of cardio-cerebrovascular and renal complications and mortality among Asian hypertensive patients.

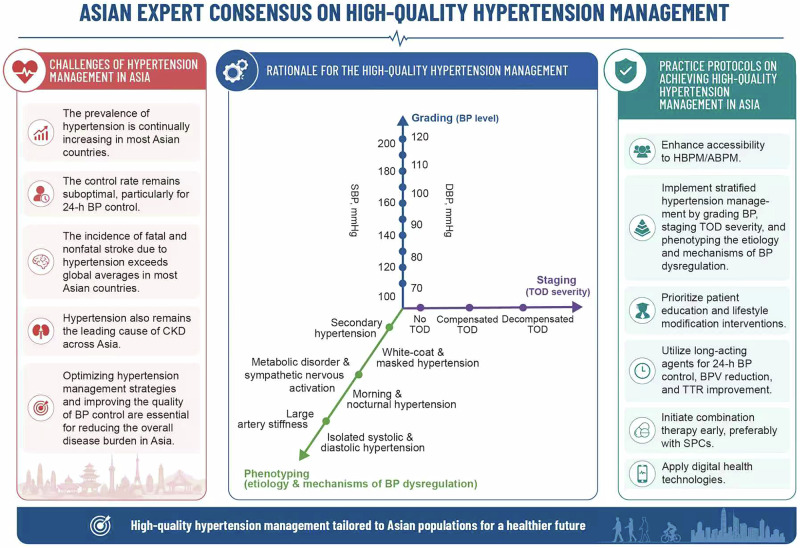

## Introduction

**Key Points:**

- Hypertension management in Asia is challenging. The prevalence of hypertension is continually increasing in most Asian countries, while the control rate remains suboptimal, particularly for 24-h (24-h) blood pressure (BP) control.
- Hypertension imposes a substantial disease burden in Asian populations. The incidence of fatal and nonfatal stroke due to hypertension exceeds global averages in most Asian countries. Hypertension also remains the leading cause of chronic kidney disease (CKD) across Asia.
- Optimizing hypertension management strategies and improving the quality of BP control are important for reducing the risk of major cardio-cerebrovascular and renal events, as well as the overall disease burden in Asia.

Hypertension management remains a major challenge in Asia. From 1990 to 2019, the total number of patients with hypertension increased by approximately 144% in the Asia-Pacific region, which was over three times the 41% increase observed in Europe and America [1]. The control rate of hypertension in most Asian countries is lower than the global average (23% for women and 18% for men), being 17% for women and 11% for men in South Asia, and 17% for women and 13% for men in East and Southeast Asia [2]^.^ Additionally, blood pressure variability (BPV) is greater in Asians than in White populations [3], and the prevalence of masked hypertension in Asia is nearly double that in Europe [4]. Several studies in the Asian population have shown that BP is also poorly controlled over 24 h, in the morning and at night [5].

In Asia overall, stroke remains the predominant complication of hypertension, while the risk of coronary heart disease (CHD) has increased substantially in the past decades. The burden of stroke and stroke-related mortality in many Asian countries ranks among the highest globally [6]. Hypertension is also a major risk factor for CKD in Asia. The mortality rates of CKD related to hypertension in Southeast Asia are among the highest worldwide [7].

There are two major challenges in the management of hypertension in Asia. One is the increasing prevalence of hypertension in most Asian countries and regions, especially in the populous countries such as China and India [8]. Another is the low control rate of hypertension in treated hypertensive patients. There are also opportunities. Out-of-office BP measurement, either ambulatory (ABPM) or home BP monitoring (HBPM), is increasingly used in Asian countries. Digital technology and artificial intelligence (AI) are also playing an increasing role in the management of chronic diseases, especially hypertension [9, 10]. We, a group of experts from several Asian countries, therefore, propose a strategy of high-quality hypertension management on the basis of out-of-office BP measurement and digital platforms to improve BP control and eventually reduce the burden of cardio-cerebrovascular and renal diseases related to hypertension.

## The concept of high-quality hypertension management

**Key Points:**

- Hypertension management according to the level of BP (grading), the severity of target organ damage (TOD, staging), and the etiology of hypertension or mechanisms of BP dysregulation (phenotyping) is important for the development of individualized treatment regimens.
- The essentials of high-quality hypertension management include optimizing 24-h and long-term BP control by using long-acting antihypertensive agents, achieving individualized hypertension treatment, reducing BPV, improving the time in target range (TTR), and eventually reducing the risk of cardio-cerebrovascular and renal complications and mortality.

**Fig. 1:**
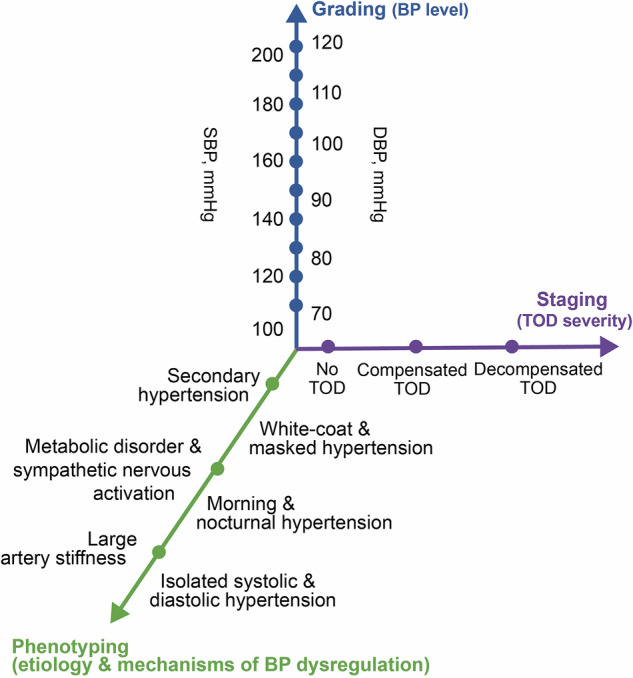
Rationale for the high-quality hypertension management according to the level of blood pressure (BP, grading), target organ damage (TOD) severity (staging) and etiology of hypertension or mechanisms of BP dysregulation (phenotyping)

Hypertension is a clinical manifestation or consequence of BP dysregulation attributed to various diseases or mechanisms. Hypertension is basically described and classified according to systolic (SBP) and diastolic BP (DBP) and impairs major perfused organs such as the brain, heart, kidneys and retina. According to the key features of hypertension, we proposed an integrated approach for the management of hypertension according to the level of BP (grading), TOD severity (staging), and etiology of hypertension or mechanisms of BP dysregulation (phenotyping) (Fig. 1).

**“Grading”** is usually based on, but far beyond, clinic or office BP, which is still the major basis for the diagnosis and treatment of hypertension in most of the current hypertension guidelines, but is apparently insufficient for the evaluation of the exposure to high BP. “Grading” should therefore include out-of-office BP measurement, either one or both of the well-evidenced ABPM and HBPM. The electronic BP measuring devices make it possible to automatically transmit the BP readings to digital platforms for storage, analysis, and feedback of the BP readings and various derived BP indices, such as BPV. With AI and deep learning, there are increasing opportunities for long-term management and control of hypertension [10].

**“Staging”** is used interchangeably with grading to classify BP in some hypertension guidelines. However, “staging” here describes the disease or TOD severity in three stages: no damage, subclinical or compensated damage, and decompensated damage or clinical complications. The “staging” reflects the current risk profiling, and is therefore different from “risk stratification”, which evaluates potential risks within a certain period of time. The latter is generally useful at the population level but is often criticized for its inaccuracy when applied to individuals. Current imaging, laboratory and hemodynamic technologies allow for not only the diagnosis of severe organ damage, that is, clinical complications, but also the very early detection of structural and functional abnormalities or malformations of major perfused organs and the vascular system. With this stage information, specific interventions and treatments can be chosen for the regression or slowing down of TOD.

**“Phenotyping”** aims to describe various diseases that cause hypertension, often referred as secondary hypertension, and mechanisms of BP dysregulation, often referred as primary hypertension, and ultimately achieve precision diagnosis, treatment or even cure of hypertension. For the so-called secondary hypertension, the goal is to systemically screen the common causes of secondary hypertension, such as primary aldosteronism, obstructive sleep apnea syndrome (OSAS), drug- and food-induced hypertension and renal parenchymal and renovascular hypertension. For the primary hypertension, the goal is to describe various mechanisms of BP dysregulation, such as arterial stiffness in the elderly, sympathetic overactivation or autonomous nervous dysfunction in middle-aged people and metabolic disorders in the young. It is now possible to treat or target the secondary causes of hypertension and mechanisms of primary hypertension as well. An intermediate step is to describe several typical phenotypes of hypertension, such as white-coat and masked hypertension, morning and nocturnal hypertension, and isolated systolic and diastolic hypertension. These phenotypes of hypertension often represent one or more mechanisms of BP dysregulation, for instance, arterial stiffness as a mechanism of isolated systolic hypertension or sympathetic overactivation as a mechanism of isolated diastolic hypertension.

High-quality hypertension management is built upon the concept of the above-mentioned integrated approach of grading the level of BP, staging the disease or TOD severity and phenotyping the etiology of hypertension or mechanisms of BP dysregulation. The ultimate goal of high-quality hypertension management is to achieve the best cardio-cerebrovascular and renal outcome by optimizing the control of office and 24-h BP, reducing BPV and improving TTR.

## Measures and strategies for the evaluation of high-quality hypertension management

### Office BP target

**Key Points:**

- Office BP target should be below 130/80 mmHg for most patients with hypertension, if tolerated.
- Structured lifestyle interventions should commence immediately as a critical component of therapy for all patients.
- For grade 1 hypertension, initial monotherapy or low-dose combination therapy should be considered.
- For grade 2 or 3 hypertension, combination therapy should be initiated immediately, preferably with single-pill combinations (SPCs).

The primary objective of antihypertensive therapy is to reduce the risk of cardio-cerebrovascular and renal complications and mortality. Based on a comprehensive assessment of hypertension grade and stage of TOD severity, intensive antihypertensive therapy should be considered to optimize BP control and minimize the risks of adverse events. Recent hypertension guidelines from various Asian countries/regions consistently recommend an office BP target of <130/80 mmHg for most hypertensive patients [11–16]. For frail or very elderly patients, a less stringent and individualized BP target is reasonable with a balance of cardiovascular benefits and vital organ perfusion.

All patients with hypertension should initiate and maintain comprehensive lifestyle interventions as a critical component of therapy, including dietary modifications, physical activity, body weight management and changes in all modifiable risk factors. For patients with grade 1 hypertension, initial monotherapy or low-dose combination therapy is recommended. For patients with grade 2 or grade 3 hypertension, combination antihypertensive therapy should be initiated immediately, preferably with SPCs. Individualized treatment strategies and office BP targets are presented in Table 1.

**Table 1:** Office blood pressure targets (mmHg) and treatment strategies by grade of hypertension and stage of target organ damage (TOD) severity

		Grade 1 (140-159/90-99 mmHg)	Grade 2 (160-179/100-109 mmHg) or Grade 3 ( ≥ 180/110 mmHg)
Office BP targets	No TOD	<140/90; < 130/80, if tolerated	<130/80
	Compensated or decompensated TOD	<130/80	
Therapeutic strategies		Monotherapy or low-dose combination	Combination therapy, preferably with SPCs

For older adults aged 65-79 years, the recommended BP target is < 140/90 mmHg, or <130/80 mmHg if well tolerated; for those aged ≥ 80 years, the recommended BP target is < 150/90 mmHg, or <140/90 mmHg if tolerated

### 24-h BP control

**Key Points:**

- Morning/nocturnal hypertension, abnormal circadian rhythm and elevated 24-h BPV are common in Asian patients.
- Comprehensive management of 24-h BP is essential for high-quality hypertension management, which includes controlling BP to targets throughout the 24-h period, reducing BPV during the day and night, and maintaining normal circadian BP rhythm.
- Standardized 24-h ABPM with validated monitors is preferred, while HBPM and wearable BP monitoring (WBPM), which are also validated, are optional for 24-h BP monitoring.
- Long-acting antihypertensive agents are prioritized for optimal pharmacokinetic stability and serve as key therapeutic tools to achieve adequate 24-h BP control.

Morning or nocturnal hypertension, non-dipping pattern and elevated 24-h BPV are common in Asian patients [3]. A key component of high-quality hypertension management is to achieve 24-h BP control, which includes lowering BP to the target throughout the 24-h period, reducing 24-h BPV, and maintaining normal circadian BP rhythm.

#### Mean of 24-h BP

The 24-h ambulatory BP is more closely associated with the risk of cardiovascular diseases and all-cause death than office BP [17, 18]. For every 20 mmHg increase in the office and 24-h SBP, the risk increased by 20% and 45%, respectively, for cardiovascular events, and by 12% and 22%, respectively, for total mortality [18].

#### Morning hypertension and morning surge

Morning BP is usually defined as home BP measurements obtained within 1 h of awakening and prior to taking medications and breakfast, or ambulatory BP readings recorded during the first 2 h after awakening. Morning hypertension is recommended to be diagnosed when morning BP is ≥135/85 mmHg, irrespective of office BP or BPs of other time periods [19]. This phenotype demonstrates particularly high prevalence among old people and patients with CKD, diabetes mellitus (especially those with autonomic neuropathy), OSAS, high sodium intake, tobacco use, alcohol consumption, metabolic syndrome, or anxiety disorders.

Morning hypertension can be generally categorized into two subtypes: “morning surge” and “sustained nocturnal and morning hypertension”, both of which are more frequently found in Asians than in Westerners [19]. These two types of morning hypertension both increase the risk of cardio-cerebrovascular and renal diseases, but may occur via different pathogenic mechanisms [19].

The HONEST (Home blood pressure measurement with Olmesartan Naive patients to Establish Standard Target blood pressure) study demonstrated that home morning BP independently predicted cardiovascular risk regardless of the office BP. Specifically, patients with office SBP < 130 mmHg but home morning SBP ≥ 145 mmHg demonstrated a 1.5-fold increase in cardiovascular risk compared to those with home morning SBP < 125 mmHg [20]^.^ The J-HOP (Japan Morning Surge-Home Blood Pressure) study further indicated the superiority of home morning SBP over office SBP in predicting stroke [21]. Moreover, analysis in hypertensive CKD patients indicated a 28% higher risk of CKD progression per 10 mmHg increase in morning SBP, independent of office BP values [22]^.^

Morning hypertension can be assessed and diagnosed by either HBPM or ABPM. Considering the limited availability of ABPM in the Asian region, and better reproducibility and closer relationship with TOD of home than ambulatory morning BP [23], morning home BP is recommended as the first target for 24-h BP control [24].

#### Nocturnal hypertension and BP circadian rhythm

Nocturnal hypertension is defined as a nighttime BP ≥ 120/70 mm Hg via ABPM or HBPM, and especially isolated nocturnal hypertension, defined as a nighttime BP ≥ 120/70 mmHg and daytime BP of <135/85 mmHg, exhibits a high prevalence in Asian populations [25]. The Chinese JingNing study reported 49.3% of 677 individuals had nocturnal hypertension in the general population [26]. This condition is particularly common in patients with CKD, with 63–73% prevalence observed in Chinese cohorts [27, 28]. Furthermore, older adults, patients with concomitant CKD, diabetes, Parkinson’s disease, sleep disorders, autonomic instability and OSAS are all associated with nocturnal hypertension [29]. Around 80% cases of morning hypertension coexist with nocturnal hypertension [17, 30].

Data from 6359 Japanese and 4088 Chinese revealed an uncontrolled nighttime BP ( ≥ 120/70 mm Hg) in 62.4% and 72.4% of patients, respectively, indicating a substantial gap in nocturnal hypertension control in Asians [31, 32]. This is probably driven by the high prevalence of salt-sensitive hypertension and excessive sodium intake in the populations. Nocturnal BP independently predicts cardiovascular and all-cause mortality better than daytime measurements [33]. Patients with isolated nocturnal hypertension exhibit elevated risks of TOD and cardiovascular events [34]. In CKD patients, elevated nocturnal SBP independently worsens cardiovascular prognosis [28]^.^

According to the nighttime BP dipping relative to the daytime BP, circadian BP rhythms can be classified into four types, including dipping (10–20% nocturnal decline), non-dipping (0–10%), reverse-dipping ( < 0%), and extreme-dipping ( > 20%). Non-dipping and reverse-dipping patterns are significantly associated with increased risks of cardiovascular mortality and TOD [17]. Pending future evidence, antihypertensive treatment might better be personalized according to circadian profiles, which include intensified nocturnal BP control for non-dippers and reverse-dippers, and cautious treatment to avoid ischemic events in extreme-dippers.

#### Short-term BPV

Short-term BPV refers to beat-to-beat, min-to-min, h-to-h and day-to-night BP fluctuations within a 24-h period. Elevated 24-h BPV in hypertensive patients correlates with increased incidence and severity of TOD [35], including left ventricular hypertrophy (LVH) [36], urinary protein excretion [37], coronary atherosclerotic plaques [38], and cerebral white matter hyperintensities [39], and independently predicts all-cause mortality [40].

Elevated 24-h BPV is prevalent in patients with acute cerebrovascular events, likely driven by transient sympathetic nervous system overactivation. Increased BPV compromises collateral perfusion and increases the risk of cerebral edema exacerbation and reperfusion injury in acute stroke. Its established prognostic value in acute ischemic stroke (AIS) includes the prediction of 90-day mortality [41], associations with severe neurological disability at 3 months, and increased risk of cardiovascular events [42]^,^ and stroke recurrence [43]. This prognostic significance extends across stroke subtypes, including large artery atherosclerosis, small arterial obstruction, and cardiogenic embolism, etc [44, 45]. Elevated 24-h BPV also predicted poor functional outcomes post-endovascular therapy [46, 47] and was validated in a registry of 28,976 AIS patients treated with thrombolysis as an independent predictor of intracerebral hemorrhage, disability, and mortality at 3 months post-rtPA (recombinant tissue Plasminogen Activator)-thrombolysis [48].

With advancing disease stages, CKD patients demonstrated a progressive increase in 24-h BPV [49, 50]. In non-dialysis CKD patients, increased short-term BPV was independently associated with TOD, including LVH, increased carotid intima-media thickness (IMT), reduced estimated glomerular filtration rate, and albuminuria [49], and predicted cardiovascular events and end-stage renal disease (ESRD) risk independent of the mean 24-h BP [51, 52]. Among hemodialysis patients, intradialytic short-term BPV was a superior predictor of cardiovascular outcomes compared with interdialytic BPV [53]. Given the unique pharmacokinetic challenges in dialysis populations, the selection of antihypertensive agents requires comprehensive pharmacokinetic evaluation, including apparent volume of distribution and dialytic clearance as well as prioritization of agents that may reduce arterial stiffness and BPV, and offer dialysis-specific benefit [54, 55]. The pathophysiological basis of elevated short-term BPV in CKD remains incompletely elucidated. Modulating BPV may confer renoprotection through enhanced renal perfusion pressure and medium-sized oxygenation-hemodynamic improvements that attenuate glomerular hyperfiltration and intrarenal hypertension, ultimately decelerating CKD progression while preserving renal function [56].

#### Strategies for 24-h BP control

The prerequisite of 24-h BP control is a comprehensive assessment of the 24-h BP profiles, including evaluations of the 24-h BP mean levels, morning and nocturnal BP patterns, and short-term BPV. Appropriate interventions should be applied to achieve optimal morning, nocturnal, and 24-h BP control, while reducing 24-h BPV and restoring a normal circadian rhythm.

##### Perform 24-h BP monitoring when feasible

ABPM remains a standard method for 24-h BP monitoring. Remote digital platforms facilitate integrated ABPM analysis and automated reporting. If possible, it is recommended that all hypertensive patients undergo a standardized ABPM at least once to confirm the diagnosis of hypertension or uncontrolled hypertension and to characterize circadian profiles. When ABPM is not possible, HBPM is recommended as a validated alternative for out-of-office BP assessment. WBPM with validated devices can also be employed, as they may provide more BP readings than conventional ABPM and give supplementary information on health metrics.

Based on current clinical evidence and expert consensus, it is recommended that a standardized ABPM be essential for the following scenarios:

1. Accurate diagnosis

- Initial hypertension diagnosis
- Characterization of the 24-h BP profiles (diurnal/nocturnal patterns)
- Detection of hypertension subtypes: morning hypertension, nocturnal hypertension, white-coat and masked hypertension

(2) Optimal treatment

- Uncontrolled office/home BP, despite optimal guideline-directed therapy
- Progressive TOD with a controlled office/home BP
- Significant BP fluctuations

(3) Post-treatment assessment

- Repeat ABPM within 3 months following treatment initiation/modification, until adequate BP control is achieved.
- Repeat ABPM within 2-3 weeks following treatment initiation/modification for high-risk populations to ensure early 24 h BP control, for instance, patients with CHD, heart failure, stroke or cerebral small vessel disease, CKD (including proteinuria and hemodialysis-dependent status), diabetes, systemic atherosclerosis, OSAS, anxiety disorders, and neurodegenerative diseases.

##### Achieve 24-h BP control

Table 2 provides ABPM thresholds that have resulted in a similar cardiovascular risk in populations corresponding to an office BP of 140/90 mmHg [57]. These thresholds are also recommended as the targets of 24-h BP control.

**Table 2:** Assessment measures and target values for 24-h BP control

Assessment	Target
24-h ambulatory BP	<130/80 mmHg
Daytime ambulatory BP	<135/85 mmHg
Nocturnal ambulatory BP	<120/70 mmHg
Morning ambulatory BP	<135/85 mmHg

For patients with suboptimal 24-h BP control, a specific management regimen is recommended as follows. The first step is to assess and correct contributing factors of hypertension, such as secondary causes and medication-related issues, including non-adherence, inappropriate dosing time, or the use of short-acting agents. On the basis of lifestyle modifications, evidence-based antihypertensive drug treatment should then be initiated. Long-acting antihypertensive agents, particularly ultra-long-acting agents with a plasma half-life of more than 24 h, are prioritized for their pharmacokinetic stability and considered key therapeutic tools to achieve adequate 24-h BP control.

##### Reduce 24-h BPV

Standard deviation (SD) and coefficient of variation (CV) are commonly used measures of BPV in 24-h ABPM reports, while SD weighted for the time intervals of day and night, and average real variability (ARV), quantifying the absolute mean difference between consecutive BP readings, are also frequently used in clinical research. Currently, evidence from interventional studies on 24-h BPV remains limited. Based on existing prospective cohort data, a 24-h SBP weighted SD ≥ 12.8 mmHg has been proposed as a marker of increased risk for cardiovascular events [35, 58], and is herein recommended as the operational cut-off value for 24-h BPV control in clinical practice. Evidence indicates that long-acting calcium channel blockers (CCB) and diuretics are more effective in reducing 24-h BPV [59, 60], and greater 24-h BPV reduction is achieved with the use of combination therapy compared to monotherapies [61, 62].

Assessment and management of 24-h BPV can be considered for patients with the following clinical conditions:

1. Severe arterial atherosclerosis (e.g., carotid or major cerebral artery stenosis >70%)
2. Stiffened artery with increased pulse pressure ( > 60 mmHg)
3. Acute or subacute stroke, or chronic stroke with residual neurological deficits, or cerebral small vessel disease
4. CKD with resistant hypertension, or advanced stage CKD, with special attention to those on hemodialysis for assessment of intradialytic and interdialytic BPV.

##### Manage 24-h BP in stroke and CKD patients

Individualized evaluation and tailored management of 24-h BP should be applied to special populations, especially for stroke and CKD patients.

After an acute stroke, significantly elevated BPV can compromise collateral circulation and increase the risk of exacerbating cerebral edema and reperfusion injury, thereby impeding neurological recovery. Continuous 24-h BP monitoring is critical to guide therapeutic optimization, preventing cerebral hypoperfusion from excessive BP fluctuations. Short-term BP target during AIS is determined by two key factors: 1) eligibility for reperfusion therapy (intravenous thrombolysis and/or endovascular thrombectomy), and 2) whether successful reperfusion has been achieved. BPV management is critical for AIS, particularly during the first 72 h of post-stroke due to heightened neurovascular instability. BPV control requires multimodal interventions addressing physiological stressors (e.g., pain, agitation). For subacute stroke patients initiating or resuming antihypertensive therapy, current evidence supports prioritizing agents with extended pharmacokinetic stability, cerebral perfusion preservation and favorable impact on 24-h BPV.

For CKD patients, management strategies diverge by renal replacement status. Non-dialysis CKD necessitates individualized dosing adjustments with regular serum electrolyte and renal function monitoring, prioritizing renin-angiotensin system inhibitors combined with long-acting CCBs to delay disease progression and reduce ESRD and cardiovascular risks. Hemodialysis-dependent CKD requires focusing on BPV during and between dialysis sessions, addressing significant intradialytic hemodynamic fluctuations through comprehensive volume assessment (emphasizing control of interdialytic weight gain <5% of dry weight; and <3% in those with recurrent intradialytic hypotension or heart failure) and cardiac function assessment to balance volume fluctuation and arterial stiffness (to improve vascular compliance), aiming to prevent intradialytic hypotension. Preferential use of low-dialyzable antihypertensive agents is necessary to manage BP elevation occurring during dialysis.

### Long-term BP management

**Key Points:**

- Long-term BP management is critical for achieving high-quality hypertension control.
- HBPM serves as the primary method for long-term BP assessment and management.
- Long term BPV and TTR can be used as key metrics for long-term BP management and are proven predictors of cardio-cerebrovascular and renal risks.
- Digital health platforms enable long-term recording of home and office BP with automated calculation of BPV indices and TTR.

The goal of high-quality hypertension management is the prevention of cardio-cerebrovascular and renal events, not exclusively BP reduction. Evidence from large-scale randomized controlled trials and observational studies demonstrates that sustained BP control during follow-up, better controlled long-term BPV, and a higher TTR are associated with reduced cardiovascular risk, irrespective of the mean level of achieved BP [63, 64]. It’s recommended to establish long-term BPV and TTR as key metrics for assessing long-term BP management.

#### Long-term BPV

Long-term BPV encompasses variations in BP occurring over week-to-week, month-to-month, quarter-to-quarter and year-to-year, including visit-to-visit BPV (vvBPV) and seasonal BP changes. vvBPV has been demonstrated to be associated with multiple TOD, adverse cardiovascular outcomes and all-cause mortality [40, 65, 66].

Patients with cerebrovascular diseases exhibit significantly higher vvBPV compared with the general population [67]. In stroke survivors, elevated vvBPV independently predicts stroke recurrence, major adverse cardiovascular events (MACE), all-cause mortality and dementia. This is robustly supported by pooled analysis of the ASCOT (Anglo Scandinavian Cardiac Outcomes Trial) and ALLHAT (Antihypertensive and Lipid-Lowering Treatment to Prevent Heart Attack Trial) studies, where stroke recurrence risk was 13.6% in the highest SBP-SD quartile versus 8.0% in the lowest quartile (*P* < 0.001) [68]. Secondary analysis of the PRoFESS (Prevention Regimen for Effectively Avoiding Second Strokes) trial further demonstrated a 15% increase in stroke recurrence, 19% increase in MACE, and 24% increase in all-cause mortality with each 10 mmHg rise in systolic vvBPV [69]. Notably, increased systolic vvBPV is also associated with dementia risk [70, 71].

Multiple studies conducted in Asian populations have demonstrated that long-term BPV is associated with CKD pathogenesis and progression. Childhood-to-adulthood long-term BPV elevation predicts adult-onset subclinical renal damage [72], while vvBPV independently accelerates renal function decline [73, 74]. Critically, systolic and diastolic vvBPV synergistically increases ESRD risk [75]. Studies involving Asian CKD patients have demonstrated that an elevated long-term BPV is significantly associated with increased risks of MACE [76], all-cause mortality [77], and dementia [78]. For hemodialysis patients, elevated vvBPV is a potent predictor of cardiovascular events, cardiovascular mortality and all-cause mortality [79–81].

#### TTR

TTR denotes the proportion of time that a patient’s BP remains within a predefined target range during follow-up. This metric reflects both long-term BP control levels and variability and can be used as a key indicator of long-term BP management efficacy. TTR can be calculated with two primary methodologies. One is direct calculation using actual measurements (expressed as the percentage of in-range measurements or days). Another is model-based estimation via linear regression interpolation between readings. TTR in clinical studies up to now has been computed with differences in target BP range, the frequency of BP measurement, and the follow-up time [82–86]. Most studies calculated TTR using office SBP, typically with a target range set at 110– 130 mmHg or 120–140 mmHg. Some studies involved upper-limit-only threshold of office SBP (130/140 mmHg) or target ranges for office DBP, home or ambulatory SBP. Follow-up time spans weeks to >1 year, with measurement frequencies ranging from daily to annual.

##### Office BP TTR

Cohort studies have established office BP-TTR as an independent predictor of cardiovascular risk beyond absolute BP levels, with higher TTR associated with lower risks in both cardiovascular and all-cause mortality [64, 87, 88]. Each 1-SD increase in TTR corresponds to a 25.4% decrease in cardiovascular event risk [89]. Post-hoc analysis of randomized controlled trials further demonstrated that systolic office BP-TTR was associated with renal outcomes, new-onset atrial fibrillation, and the risk of suspected dementia. A retrospective, cohort study of participants in SPRINT (Systolic Blood Pressure Intervention Trial) and the ACCORD BP trial (Action to Control Cardiovascular Risk in Diabetes–Blood Pressure) showed that the risk of major adverse kidney events declined when TTR increased from 0% to 43%, then plateaued until TTR exceeded 73%, after which the renal risk further decreased. This observation suggested that lowering the risk of adverse kidney outcomes might be achievable through extremely strict SBP control ( > 70% TTR) [83]. Analysis of 9552 hypertensive participants in the Chinese Kailuan cohort revealed that using an intensive (110–130 mmHg) versus a conventional SBP target (120–140 mmHg) was associated with more cardiovascular risk reduction, and this benefit progressively amplified over time [85].

##### Home BP TTR

The predictive value of SBP-TTR for cardiovascular events has also been investigated through HBPM. In the J-HOP extension study (mean follow-up of 6.3 ± 3.8 years), home SBP-TTR (SBP target range 100–135 mmHg) was calculated in subjects who measured their home SBP for ≥5 days during a 13-day baseline period. Results demonstrated that every 10% reduction in home SBP-TTR was associated with a 4% increase in total cardiovascular events risk (*p* = 0.033) and a 9% increase in stroke risk (*p* = 0.004). The findings also indicated that maintaining home SBP-TTR ≥ 67% was required for effective stroke risk reduction [90].

##### Ambulatory BP TTR

In a person-level meta-analysis of 14,230 individuals enrolled in 14 population cohorts, systolic and diastolic ambulatory BP were combined to assess 24-h, daytime, and nighttime TTR with thresholds for non-elevated ambulatory BP set at <115/65, <120/70, and <110/60 mmHg, respectively. Over 10.9 years, deaths and cardiovascular endpoints decreased across increasing 24-h TTR quartiles. Analyses of daytime and nighttime TTR in relation to cardiovascular mortality, coronary endpoints and stroke produced confirmatory results [91].

In a prospective multicenter follow-up study of 228 AIS patients, 24-h TTR was 35% for SBP (90–140 mmHg) and 64% for DBP (60–90 mmHg) during the first 72 h post-stroke onset. In 175 patients without prior disability, an increase in 24-h TTR of DBP was significantly associated with a decreased risk of disability/death (hazard ratio 0.96, 95% CI 0.95-0.99, *p* = 0.007). These findings underscore the necessity of 24-h TTR assessment in AIS management [82].

#### Strategies for long-term BP management

##### Perform HBPM

While current evidence on long-term BPV and TTR primarily originates from studies with office BP measurements, HBPM offers greater feasibility and may now serve as the primary approach for long-term BP monitoring and management. All patients should be trained to perform standardized HBPM regularly using validated upper-arm BP devices. Considering the circadian BP variations, it is recommended to perform HBPM in consistent daily time windows (e.g., morning and evening), particularly when used to calculate long-term BPV and TTR. For hemodialysis patients, HBPM is critically important, as home BPV can partly reflect volume fluctuations and home TTR can better indicate volume control status and cardiac function.

##### Reduce long-term BPV

To manage long-term BPV, clinicians need an operational threshold. vvBPV is the core indicator for evaluating long-term BPV. Population-based studies have proposed a specific threshold for elevated vvBPV. The ASCOT Legacy study demonstrated that hypertensive patients had significantly increased cardiovascular risk when the vvBPV (SBP SD) ≥ 13 mmHg [92]. Additionally, vvBPV (SBP SD) ≥ 15.6 mmHg identified patients at increased risks of total mortality, CHD, stroke, and ESRD [93]. Though interventional benefits and optimal targets warrant further investigation, vvBPV (SBP SD) < 13 mmHg may represent a reasonable threshold associated with non-elevated cardiovascular risk. Various antihypertensive drug classes may have different effects on long-term BPV, with evidence from ASCOT, ALLHAT and SPRINT suggesting that long-acting CCBs induce the most effective long-term BPV lowering [94–96].

All hypertensive patients are recommended to undergo vvBPV assessment through standardized office BP measurements during follow-up. Regular standardized HBPM or WBPM can help the evaluation of long-term BPV and therefore enhance cardiovascular risk stratification and prediction. Patients with the following conditions are preferably suggested to evaluate and reduce long-term BPV:

1. Older adults ( ≥ 65 years) with vascular stiffening.
2. Patients with ischemic comorbidities (coronary artery disease, renal artery stenosis, large-artery atherosclerotic stroke) warrant close long-term BPV monitoring as hemodynamic fluctuations may compromise perfusion and increase adverse event risk.
3. Patients with cerebral small vessel disease, particularly those exhibiting neuroimaging markers (white matter hyperintensities, lacunar infarcts, cerebral microbleeds, or perivascular spaces), require stringent long-term BPV control to mitigate dementia risk and disease progression.
4. Hemodialysis patients should be monitored for both intradialytic short-term BPV and interdialytic vvBPV, supplemented by HBPM where feasible, to optimize treatment regimens and mitigate cardiovascular and mortality risk.
5. In patients with elevated BPV attributable to secondary causes, the underlying etiologies—such as pheochromocytoma or menopausal factors in women—should be identified and appropriately treated.

##### Improve TTR

Calculating TTR requires a long-term recording of BP data and related calculations. Therefore, digital health tools (e.g., electronic BP diaries or mobile applications) are recommended for the computation of TTR. A retrospective longitudinal study demonstrated that long-term continuous use of a long-acting drug-based regimen significantly improved SBP-TTR over three years [97, 98].

Key management strategies to improve TTR include:

1. Current evidence primarily supports SBP of 120–140 mmHg as a target range for office BP management. Patients with specific comorbidities may need an individualized target BP range.
2. Up to now, there is no universally established threshold for TTR. Most studies have employed a quartile-based grouping approach for analysis, suggesting that TTR over 75% is associated with significant clinical benefit. Therefore, TTR ≥ 75% may be considered an acceptable threshold.
3. Long-term sufficient antihypertensive therapy based on long-acting antihypertensive agents is foundational to improving TTR.

## Practice protocols on achieving high-quality hypertension management in the Asian population

**Key Points:**

- Enhance accessibility to out-of-office BP measurement and promote HBPM/ABPM
- Implement stratified hypertension management by grading BP, staging TOD severity, and phenotyping the etiology of hypertension or mechanisms of BP dysregulation
- Prioritize patient education and lifestyle modification interventions
- Utilize long-acting agents for 24-h BP control, BPV reduction, and TTR improvement
- Initiate combination therapy early to optimize BP control and adherence
- Apply digital health technologies (Internet, wearable devices and artificial intelligence technology) for high-quality management

In 2022, The HOPE Asia Network proposed a seven-action approach for hypertension management in Asia [24]. Incorporating recent evidence, we propose the following key practice points to achieve high-quality hypertension management in Asian populations.

### Comprehensive and accurate BP measurement

Sustained public education campaigns should be initiated. Validated BP monitors should be provided in hospitals and public locations to improve BP measurement accessibility. Real-time hypertension consultation services should be offered via online health platforms following BP measurements. Out-of-office BP measurement should be performed. To facilitate BP measurement at home, this consensus document advocates “One home, one device, regular measurement”. ABPM should be implemented when available.

### Stratified hypertension management by grading BP, staging TOD severity, and phenotyping the etiology of hypertension or mechanisms of BP dysregulation

#### 1. Tailor antihypertensive therapy according to the level of BP (grading)

It begins with determining the appropriate magnitude of BP reduction, which is calculated as the difference between the patient’s baseline level and the individualized BP target. In parallel, an individualized time-to-target, typically ranging from 4 to 12 weeks, should be established based on treatment tolerance and clinical characteristics, allowing for gradual adjustment and minimizing the risk of adverse events.

#### 2. Personalized management according to the TOD severity (staging)

1) Comprehensive risk factor intervention: smoking cessation, weight reduction, glycemic control, lipid management, management of mental and psychological disorders, and improvement of sleep quality; 2) target organ protection: assess subclinical TOD, e.g., LVH, albuminuria, increased IMT, and arterial stiffness; 3) clinical complication management: treat established conditions, e.g., atrial fibrillation, cardiac/renal insufficiency, and stroke.

#### 3. Indications for etiological subtyping and phenotyping (phenotyping):

1) Hypertension onset at age <30 years (including pediatric cases); 2) sudden-onset or aggravation of hypertension in previously controlled patients; 3) true resistant hypertension; 4) hypertension emergencies; 5) grade 3 hypertension (BP ≥ 180/110 mmHg); 6) severe and/or TOD disproportionate to BP history; 7) the presence of biochemical/clinical features of endocrine or renal parenchymal or renovascular hypertension; 8) hypertension in pregnancy. Patients with suspected secondary hypertension require immediate referral to specialists for standardized diagnostic evaluation and targeted therapy.

### Patient education and lifestyle modifications

BP reduction is achievable through sustained lifestyle modifications, including dietary optimization (notably salt restriction to <5 g/day), smoking cessation, regular physical activity, sleep improvement, increased potassium intake, alcohol restriction, and psychological well-being maintenance. These non-pharmacological interventions constitute an integral component of hypertension management throughout its entire course.

### Use of long-acting agents for 24-h BP control, BPV reduction, and TTR improvement

Long-acting antihypertensive agents should be prioritized to achieve superior 24-h BP control, including morning surge attenuation and nocturnal BP management. Given the evidence that certain long-acting antihypertensive agents can effectively reduce BPV and improve TTR [59–62, 94–98], these regimens, whether used as monotherapy or in combination, are preferred as core therapeutic options to achieve high-quality hypertension management.

### Early combination therapy to optimize BP control and adherence

To enhance medication adherence, these evidence-based strategies should be implemented: 1) prioritize early initiation of combination therapy using SPCs, 2) promote standardized HBPM with a validated device, 3) establish collaborative care partnerships through family-inclusive education and shared decision-making, and 4) integrate patient-acceptable digital adherence tracking technologies.

### Digital platforms for high-quality hypertension management

Validated smart wearable BP monitors may help enhance hypertension detection and control rates. For patients with established hypertension, regular use facilitates comprehensive assessment of diurnal BP variations, informs clinical decision-making, and improves long-term control—ultimately reducing cardiovascular risk [99].

Standardized transmission and analysis of HBPM and ABPM data through integrated informatics platforms optimize the clinical utilization of out-of-office BP monitoring. By combining office and ambulatory measurements, clinicians can achieve more precise hypertension phenotyping, conduct a comprehensive assessment of circadian rhythm, calculate TTR, and evaluate BPV.

## Summary

In this consensus document, we propose a high-quality hypertension management framework tailored to the epidemiological characteristics of Asian populations and regional prevention challenges, by advocating comprehensive 24-h ABPM and long-term HBPM, using BPV and TTR as core quality metrics, and promoting the use of long-acting antihypertensive agents, at full dose or in combination to achieve 24-h and long-term BP control, BPV reduction and TTR improvement. Special attention should also be given to the management of hypertensive patients with CKD and stroke.

These evidence-based hypertension management strategies will help in improving hypertension control across Asia, reducing the risk of cardio-cerebrovascular and renal complications, and ultimately alleviating the disease burden and mortality attributable to hypertension in the region.
